# Psychological impact of COVID-19 on healthcare workers: cross-sectional analyses from 14 countries

**DOI:** 10.1017/gmh.2022.35

**Published:** 2022-07-08

**Authors:** Sherief Ghozy, Wendy M. Cross, Shariful Islam, Adhra Hilal Al-Mawali, Alaa Ashraf AlQurashi, Amr Hamza, Bindu Joseph, Biswajit Banik, Deena H. Elsori, Farhana Sultana, Farhana Yasmin, Ilias Mahmud, Louisa Lam, Majeda Hammoud, Masudus Salehin, Mohammed Ali Keblawi, Nael Kamel Eltewacy, Nahed Al Laham, Nashwa El-Khazragy, Natalia Oli, Patraporn Tungpunkom, Sami Almustanyir, Sek Ying Chair, Sheikh M. Alif, Sondos Al-Madhoun, Wai Tong Chien, Muhammad Aziz Rahman

**Affiliations:** 1 Neuroradiology Department, Mayo Clinic, Rochester, MN, USA; 2 Institute of Health and Wellbeing, Federation University Australia, Berwick, Victoria, Australia; 3 Institute for Physical Activity and Nutrition (IPAN), Faculty of Health, Deakin University, Burwood, Victoria, Australia; 4 Ministry of Health, Muscat, Sultanate of Oman; 5 Research Center, King Fahad Medical City, Riyadh, Kingdom of Saudi Arabia; 6 Faculty of Medicine, University of Aleppo, Aleppo, Syria; 7 Abu Dhabi University, Abu Dhabi, United Arab Emirates with; 8 Dean's Office, Rabdan Academy, Abu Dhabi, United Arab Emirates; 9 Telstra Health, Melbourne, Victoria, Australia; 10 Department of English Language and Literature, Lahore Garrison University, Lahore, Pakistan; 11 College of Public Health and Health Informatics, Qassim University, Qassim, Kingdom of Saudi Arabia; 12 Faculty of Medicine, Kuwait University, Kuwait, Kuwait; 13 Faculty of Pharmacy, Beni-Suef University, Minia, Egypt; 14 Faculty of Applied Medical Sciences, Al Azhar University-Gaza, Gaza Strip, Palestine; 15 Faculty of Medicine, Ain Shams University, Cairo, Egypt; 16 Department of Community Medicine, Kathmandu Medical College, Kathmandu, Nepal; 17 Faculty of Nursing, Chiang Mai University, Chiang Mai, Thailand; 18 Ministry of Health, Riyadh, Kingdom of Saudi Arabia; 19 The Nethersole School of Nursing, The Chinese University of Hong Kong, Shatin, New Territories, Hong Kong; 20 School of Public Health and Preventive Medicine, Monash University, Clayton, Victoria, Australia; 21 School of Population and Global Health, The University of Melbourne, Melbourne, Victoria, Australia; 22 Department of Noncommunicable Diseases, Bangladesh University of Health Sciences (BUHS), Dhaka, Bangladesh; 23 Faculty of Public Health, Universitas Airlangga, Surabaya, Indonesia

**Keywords:** Coronavirus, COVID-19, healthcare workers, mental health, psychological distress

## Abstract

**Background:**

Healthcare workers (HCWs) have been impacted psychologically due to their professional responsibilities over the prolonged era of the coronavirus disease 2019 (COVID-19) pandemic. The study aimed to identify the predictors of psychological distress, fear, and coping during the COVID-19 pandemic among HCWs.

**Methods:**

A cross-sectional online survey was conducted among self-identified HCWs across 14 countries (12 from Asia and two from Africa). The Kessler Psychological Distress Scale, the Fear of COVID-19 Scale, and the Brief Resilient Coping Scale were used to assess the psychological distress, fear, and coping of HCWs, respectively.

**Results:**

A total of 2447 HCWs participated; 36% were doctors, and 42% were nurses, with a mean age of 36 (±12) years, and 70% were females. Moderate to very-high psychological distress was prevalent in 67% of the HCWs; the lowest rate was reported in the United Arab Emirates (1%) and the highest in Indonesia (16%). The prevalence of high levels of fear was 20%; the lowest rate was reported in Libya (9%) and the highest in Egypt (32%). The prevalence of medium-to-high resilient coping was 63%; the lowest rate was reported in Libya (28%) and the highest in Syria (76%).

**Conclusion:**

COVID-19 has augmented the psychological distress among HCWs. Factors identified in this study should be considered in managing the wellbeing of HCWs, who had been serving as the frontline drivers in managing the crisis successfully across all participating countries. Furthermore, interventions to address their psychological distress should be considered.

## Introduction

The impact of the coronavirus disease 2019 (COVID-19) pandemic was diverse and had impacted physical, psychological, economic, and social contexts globally (Álvarez-Iglesias *et al*., 2021; Zheng *et al*., 2021). Some populations were at higher risk of facing those impacts. For instance, healthcare workers (HCWs) were more vulnerable to contracting the infection than others due to the nature of their job responsibilities. That posed a significant threat not only on their health and wellbeing, but that of their co-workers, as well as the risk on their family members (Chou *et al*., 2020). The International Council of Nurses (ICN) reported deaths of many hundreds of nurses from COVID-19 worldwide. Therefore, working with patients from a high risk of infection areas could lead to mental health problems, including stress, anxiety, and depression (ICN, 2020). Studies demonstrated that the psychological wellbeing of HCWs had been considerably impacted due to their additional efforts to manage the high volume of COVID-19 patients during the pandemic (Chew *et al*., 2020; Kang *et al*., 2020; Sirois and Owens, 2020). Such additional stress of the pandemic on their usual work-related stress was often considered part of the routine responsibilities. Importantly, during the pandemic many HCWs were required to be redeployed to work in settings outside of their clinical expertise/specialty. They had to work extra shifts or longer hours (Shechter *et al*., 2020), often with lack of resources, inadequate protection, and amidst high risk of infection (Alizadeh *et al*., 2020). If the existing stress and COVID-19-related psychological distress were sustained, those could have impacted health and wellbeing of HCWs (Shechter *et al*., 2020), potentially leading to many short-and long-term mental health consequences. Some of the documented adverse events in healthcare settings included suicide, substance abuse, intention to quit their job, reduced provision of quality of care to patients following cognitive impairment and work-related stress, and being irritable with colleagues (Taylor *et al*., 2007; Wu *et al*., 2008; Dall'Ora *et al*., 2015; Adler *et al*., 2017). Therefore, it was imperative to understand the factors contributing to the risk of developing psychological distress during the pandemic, which could be considered while formulating policies in healthcare settings to reflect on better interventions for that critical workforce.

The impact of COVID-19 varied across the globe, so were the subsequent responses at a country level due to wide variations in health systems delivery. Psychological distress among HCWs was documented in earlier studies that showed very high and exaggerated impact during the COVID-19 pandemic compared to usual, ordinary circumstances and the general population (Lai *et al*., 2020; Robles *et al*., 2020). However, most of those studies were country-specific, recruiting individuals from a single country. Since the impact of COVID-19 varied across the globe, subsequent responses from health authorities also varied due to wider variations in health systems, quality of clinical care, and health systems delivery mechanisms across countries. While in some countries, healthcare resources were not overwhelmed during the pandemic, in others, they were over-burdened (Dobson *et al*., 2021). A thorough understanding of the psychological burden among HCWs was vital during that critical juncture of the pandemic (Kafle *et al*., 2021). Hence, it was justified to examine the psychological impact of the pandemic on HCWs across multiple countries. Our previous global study showed that doctors had higher psychological distress but lower levels of fear, whereas nurses had high resilient coping during the COVID-19 pandemic (Rahman *et al*., 2021*a*). However, factors associated with such psychological distress, fear, and coping among HCWs were not examined in that study (Rahman *et al*., 2021*a*). Therefore, we aimed to identify the predictors of psychological distress, fear, and coping among HCWs across multi-country settings.

## Methods

### Study design and population

This cross-sectional online survey included data of self-identified HCWs from 14 countries (12 from Asia and two from Africa), including China (Hong Kong), Egypt, Indonesia, Jordan, Kuwait, Libya, Nepal, Oman, Pakistan, Palestine, Saudi Arabia, Syria, Thailand, and the United Arab Emirates (UAE), which was extracted from the global study led by the last author (MAR) (Rahman *et al*., 2021*a*).

### Study population and sampling

HCWs who have completed the online questionnaire in any of the proposed languages (English/Arabic/Thai/Nepali) from those selected countries were included in this analysis. First, an affirmative response in the survey determined their identities as HCWs: ‘Do you identify yourself as a health care worker?’ Then the requested responses were ‘doctors’, or, ‘nurses’, or ‘other health care workers’.

### Sampling and data collection

Our previous study detailed sample size calculation and data collection procedures (Rahman *et al*., 2021*a*). Data were collected using an online questionnaire, which was distributed to the target population through Google form. The timeframe was November 2020 to January 2021. All responses were voluntary and anonymous; no incentives were offered to complete the questionnaire.

### Study tool

The study questionnaire was translated to all of the aforementioned languages. The responses were translated back to English again under the supervision of local team leaders of each country, where responses were obtained and recorded for analyses in the current study. The study tools were described in prior published studies (Rahman *et al*., 2020, 2021*a*, 2021*b*; Bahar Moni *et al*., 2021; Chair *et al*., 2021), and their reliability was also assessed (Rahman *et al*., 2021*c*). The Kessler Psychological Distress Scale (K-10), the Fear of COVID-19 Scale (FCV-19S), and the Brief Resilient Coping Scale (BRCS) were used to assess psychological distress, fear, and coping of HCWs, respectively (Furukawa *et al*., 2003; Sinclair and Wallston, 2004; Ahorsu *et al*., 2020). Based on the scoring in each tool, psychological distress was categorized into low (10–15) and moderate-to-very high (16–50), fear into low (7–21) and high (22–35), and coping into low (4–13) and medium-to-high (14–20).

### Data analysis

Data were analysed using R software version 4.1.1. Means and standard deviations (±s.d.) were reported for continuous variables; frequencies and percentages for categorical variables. Both univariate and multivariate analyses were conducted to assess factors associated with fear, distress, and coping strategies. Potential confounders were adjusted in the multivariate analyses. A *p* value < 0.05 was used as a cut-off for statistical significance. The findings were presented as odds ratios (ORs), adjusted odds ratios (AORs) with 95% confidence intervals (CIs). Furthermore, country-wise analyses were conducted to compare the outcomes between participant countries. All the countries were organized according to severity, starting with countries with the lowest prevalence rates of medium-to-high levels of psychological distress, high levels of fear, and medium-to-very high levels of coping.

## Results

### Characteristics of HCWs

A total of 2447 HCWs were included in this study; 887 (36.2%) were doctors, 1032 (42.2%) were nurses, and 528 (21.6%) were others. The mean (±s.d.) age was 36.1 (±11.7) years, and the majority (70.2%) were females. Most participants were from Indonesia (16.4%), Thailand (12.5%), Hong Kong (11.5%), Jordan (10.3%), and Saudi Arabia (10.1%). Most of them (81%) self-identified as frontline/essential service workers. Most of the HCWs mentioned that their jobs were impacted by COVID-19 (79.7%); however, only 38.9% perceived moderate to great distress. Most participants said that they had no comorbidities (72.8%), were never smoked (85.3%), and did not drink alcohol within the past 4 weeks before data collection (89.8%). Direct contact with suspected/known COVID-19 cases was reported by 753 HCWs (31.2%), and 274 (11.5%) HCWs tested positive for COVID-19. A third of the HCWs (36%) visited a healthcare provider within the last 6 months, and one in 10 participants (13.9%) used healthcare services to address COVID-19-related stress. The detailed characteristics of the participants are presented in Table 1.

**Table 1.** Characteristics of the study population

Characteristics	Total, *n* (%)
Total study participants	2447
Age (in years)	2159
Mean (±s.d.)	36.1 (11.7)
Age groups	2159
18–29 years	783 (36.3)
30–59 years	1287 (59.6)
⩾60 years	89 (3.6)
Gender	2435
Male	726 (29.8)
Female	1709 (70.2)
Country of residence	2447
Egypt	90 (3.7)
Hong Kong	282 (11.5)
Indonesia	401 (16.4)
Jordan	252 (10.3)
Kuwait	149 (6.1)
Libya	80 (3.3)
Nepal	119 (4.9)
Oman	155 (6.3)
Pakistan	63 (2.6)
Palestine	95 (3.9)
Saudi Arabia	247 (10.1)
Syria	173 (7.1)
Thailand	307 (12.5)
UAE	34 (1.4)
Born in the same country of residence	2419
No	311 (12.9)
Yes	2108 (87.1)
Living status	2420
Live without family members	368 (15.2)
Live with family members	2052 (84.8)
Highest educational/vocational qualification	2423
Primary/Grade 1 to 6	5 (0.2)
Secondary/Higher Secondary/Grade 7 to 12	91 (3.8)
Certificate/Diploma/Trade qualifications	238 (9.8)
Bachelor/Masters/PhD	2089 (86.2)
Current employment condition	2394
Jobs affected by COVID-19 (lost job/working hours reduced/afraid of job loss)	1907 (79.7)
Have an income source (employed/Government benefits)	487 (20.3)
Perceived distress due to change of employment status	2357
A little to none	1439 (61.1)
Moderate to a great deal	918 (38.9)
Improved working situation due to change of employment situation	2345
A little to none	1734 (73.9)
Moderate to a great deal	611 (26.1)
Self-identification as a frontline or essential service worker	2447
No	465 (19.0)
Yes	1982 (81.0)
Types of HCWs	2447
Doctors	887 (36.2)
Nurses	1032 (42.2)
Others	528 (21.6)
COVID-19 impacted financial situation	2447
No impact	1096 (44.8)
Yes, impacted positively	306 (12.5)
Yes, impacted negatively	1045 (42.7)
Co-morbidities	2446
No	1780 (72.8)
Mental health issue	66 (2.7)
Other co-morbidity	600 (24.5)
Co-morbidities	2446
No	1780 (72.8)
Single co-morbidity	494 (20.2)
Multiple co-morbidities	172 (7.0)
Smoking	2447
Never smoked	2088 (85.3)
Ever smoker (daily/non-daily/Ex)	359 (14.7)
Increased smoking over the last 6 months	268
No	131 (48.9)
Yes	137 (51.1)
Current alcohol drinking (last 4 weeks)	2405
No	2159 (89.8)
Yes	246 (10.2)
Increased alcohol drinking over the last 6 months	246
No	179 (72.8)
Yes	67 (27.2)
Contact with known/suspected case of COVID-19	2411
No	1021 (42.3)
Unsure	248 (10.3)
Yes, had indirect contact	389 (16.1)
Yes, provided direct care	753 (31.2)
Experience related to COVID-19 pandemic (multiple responses possible)	2377
No known exposure to COVID-19	1603 (67.4)
Tested positive for COVID-19	274 (11.5)
Tested negative for COVID-19 by self-isolated	461 (19.4)
Had recent overseas travel history and was in quarantine	39 (1.6)
Self-identification as a patient (visited a healthcare provider in the last 6 months)	2410
No	1542 (64.0)
Yes	868 (36.0)
Healthcare service use in the last 6 months	781
In-person visit to a healthcare provider	593 (75.9)
Telehealth consultation/use of national helpline	143 (18.3)
Used both services	45 (5.8)
Perceived mental health status	2447
Poor to fair	616 (25.2)
Good to excellent	1831 (74.8)
Healthcare service use to overcome COVID-19-related stress in the last 6 months	2403
No	2068 (86.1)
Yes	335 (13.9)
Type of healthcare service used to overcome COVID-19-related stress in the last 6 months	2129
Consulted a GP	99 (31.1)
Consulted a psychologist	15 (4.7)
Consulted a psychiatrist	20 (6.3)
Used specialized mental healthcare settings	10 (3.1)
Used mental health resources	19 (6.0)
Used mental health resources available through media	60 (18.9)
Used mental health support services	26 (8.2)
Used combination of services	69 (21.7)

### Levels of psychological distress, fear of COVID-19, and resilient coping

Regarding the levels of psychological distress, the mean K-10 score was 20.9 (± 8.6). The prevalence of low, moderate, high, and very high levels of psychological distress was 32.8, 27.9, 22.4, and 17.0%, respectively. The different domains of the K-10 score are presented in Table 2. Regarding the levels of fear, the mean FCV-19S score was 16.3 (± 6.1). The prevalence of low and high levels of fear of COVID-19 was 80.1% and 19.9%, respectively. The different domains of the FCV-19S score are presented in Table 3. The mean BRCS score was 14.2 (± 2.9), while the prevalence of low, moderate, and high resilient coping was 37, 47, and 16.1%, respectively. The different domains of the BRCS score are presented in Table 4.

**Table 2.** Level of psychological distress among the study participants

K-10 items	Total, *n* (%)
About how often did you feel tired out for no good reason?	2447
None	517 (21.1)
A little of the time	700 (28.6)
Some of the time	844 (34.5)
Most of the time	299 (12.2)
All of the time	87 (3.6)
About how often did you feel nervous?	2447
None	486 (19.9)
A little of the time	843 (34.5)
Some of the time	726 (29.7)
Most of the time	297 (12.1)
All of the time	95 (3.9)
About how often did you feel so nervous that nothing could calm you down?	2447
None	1134 (46.3)
A little of the time	601 (24.6)
Some of the time	500 (20.4)
Most of the time	150 (6.1)
All of the time	62 (2.5)
About how often did you feel hopeless?	2447
None	1123 (45.9)
A little of the time	666 (27.2)
Some of the time	460 (18.8)
Most of the time	137 (5.6)
All of the time	61 (2.5)
About how often did you feel restless or fidgety?	2447
None	808 (33.0)
A little of the time	713 (29.1)
Some of the time	638 (26.1)
Most of the time	224 (9.2)
All of the time	64 (2.6)
About how often did you feel so restless you could not sit still?	2447
None	1271 (51.9)
A little of the time	607 (24.8)
Some of the time	404 (16.5)
Most of the time	128 (5.2)
All of the time	37 (1.5)
About how often did you feel so depressed?	2447
None	998 (40.8)
A little of the time	773 (31.6)
Some of the time	462 (18.9)
Most of the time	152 (6.2)
All of the time	62 (2.5)
About how often did you feel that everything was an effort?	2447
None	597 (24.4)
A little of the time	758 (31.0)
Some of the time	658 (26.9)
Most of the time	319 (13.0)
All of the time	115 (4.7)
About how often did you feel so sad that nothing could cheer you up?	2447
None	1102 (45.0)
A little of the time	665 (927.2)
Some of the time	448 (18.3)
Most of the time	157 (6.4)
All of the time	75 (3.1)
About how often did you feel worthless?	2447
None	1408 (57.5)
A little of the time	529 (21.6)
Some of the time	337 (13.8)
Most of the time	107 (4.4)
All of the time	66 (2.7)
K10 score (total)	2447
Mean (±s.d.)	20.9 (8.6)
Level of psychological distress (K10 categories)	2447
Low (score 10–15)	802 (32.8)
Moderate (score 16–21)	682 (27.9)
High (score 22–29)	548 (22.4)
Very high (score 30–50)	415 (17.0)

**Table 3.** Level of fear of COVID-19 among the study participants

FCV-19S items	Total, *n* (%)
I am most afraid of COVID-19	2446
Strongly disagree	413 (16.9)
Disagree	522 (21.3)
Neither agree nor disagree	615 (25.1)
Agree	695 (28.4)
Strongly agree	201 (8.2)
It makes me uncomfortable to think about COVID-19	2446
Strongly disagree	445 (18.2)
Disagree	524 (21.4)
Neither agree nor disagree	543 (22.2)
Agree	796 (32.5)
Strongly agree	138 (5.6)
My hands become clammy when I think about COVID-19	2446
Strongly disagree	1369 (56.0)
Disagree	601 (24.6)
Neither agree nor disagree	282 (11.5)
Agree	174 (7.1)
Strongly agree	20 (0.8)
I am afraid of losing my life because of COVID-19	2446
Strongly disagree	751 (30.7)
Disagree	550 (22.5)
Neither agree nor disagree	496 (20.3)
Agree	478 (19.5)
Strongly agree	171 (7.0)
When watching news and stories about COVID-19 on social media, I become nervous or anxious	2446
Strongly disagree	586 (24.0)
Disagree	511 (20.9)
Neither agree nor disagree	502 (20.5)
Agree	715 (29.2)
Strongly agree	132 (5.4)
I cannot sleep because I'm worrying about getting COVID-19	2446
Strongly disagree	1373 (56.1)
Disagree	551 (22.5)
Neither agree nor disagree	335 (13.7)
Agree	159 (6.5)
Strongly agree	28 (1.1)
My heart races or palpitates when I think about getting COVID-19	2446
Strongly disagree	179 (7.3)
Disagree	187 (7.6)
Neither agree nor disagree	919 (37.6)
Agree	940 (38.4)
Strongly agree	221 (9.0)
FCV-19S score (total)	2446
Mean (±s.d.)	16.3 (6.1)
Level of fear of COVID-19 (FCV-19S categories)	2446
Low (score 7–21)	1960 (80.1)
High (score 22–35)	486 (19.9)

**Table 4.** Coping during COVID-19 pandemic among the study participants

BRCS items	Total, *n* (%)
I look for creative ways to alter difficult situations	2446
Does not describe me at all	179 (7.3)
Does not describe me	187 (7.6)
Neutral	919 (37.6)
Describes me	940 (38.4)
Describes me very well	221 (9.0)
Regardless of what happens to me, I believe I can control my reaction to it	2446
Does not describe me at all	95 (3.9)
Does not describe me	194 (7.9)
Neutral	735 (30.0)
Describes me	1119 (45.7)
Describes me very well	303 (12.4)
I believe I can grow in positive ways by dealing with difficult situations	2446
Does not describe me at all	58 (2.4)
Does not describe me	106 (4.3)
Neutral	599 (24.5)
Describes me	1311 (53.6)
Describes me very well	372 (15.2)
I actively look for ways to replace the losses I encounter in life	2446
Does not describe me at all	84 (3.4)
Does not describe me	154 (6.3)
Neutral	809 (33.1)
Describes me	1107 (45.2)
Describes me very well	292 (11.9)
BRCS score (total)	2446
Mean (±s.d.)	14.2 (2.9)
Level of coping (BRCS categories)	2446
Low resilient coping (score 4–13)	904 (37.0)
Medium resilient coping (score 14–16)	1149 (47.0)
High resilient coping (score 17–20)	393 (16.1)

### Predictors of psychological distress, fear, and coping

In the adjusted model, moderate to very-high psychological distress was associated with being, living with family members, perceived moderate to a great deal of distress, having comorbid conditions other than mental health issues, history of smoking, increased alcohol consumption within the last 6 months, unsure and indirect contact with known/suspected cases of COVID-19, self-identification as a patient (in addition to being a HCW), having high levels of fear related to COVID-19, and using healthcare services to overcome COVID-19-related stress. Conversely, low levels of psychological distress was associated with being aged ⩾30 years, self-identification as a nurse, having been negatively impacted finances due to COVID-19, perceived good to excellent status of own mental health (online Supplementary Table S1).

Higher levels of fear of COVID-19 were associated with being aged 30–59 years, females, perceived moderate to a great deal of distress related to the employment situation, having other comorbid conditions other than mental health issues, current alcohol consumption, unsure contact with known/suspected COVID-19 cases, having moderate to very-high levels of psychological distress, and using healthcare services to overcome COVID-19-related stress. On the other hand, lower levels of fear were associated with perceived own mental health as good to excellent, visiting a healthcare provider in the last 6 months, and having medium to high resilient coping (online Supplementary Table S2).

Higher levels of coping was associated with being aged 30–59 years, negatively impacted financially due to COVID-19, perceived mental health as good to excellent, and providing direct care to known/suspected COVID-19 cases. However, lower levels of resilient coping were associated with having psychiatric or mental issues, self-isolation (despite negative test results for COVID-19), and having high levels of fear of COVID-19 (online Supplementary Table S3).

### Psychological distress, fear, and coping per country

By country-wise analysis, the prevalence of moderate to very high psychological distress was variable within the participating countries, the lowest being in the UAE (1.4%) and the highest in Indonesia (16.4%). Compared to the UAE, the levels of psychological distress were significantly lower in Pakistan in the multivariate analyses. High levels of fear also varied across countries, being lowest in Libya (8.8%) and highest in Egypt (32.2%). Levels of fear among HCWs were found significantly higher in Oman, Hong Kong, and Pakistan when compared with the baseline country, Libya. Similarly, moderate to high coping scores varied across countries, the lowest being in Libya (27.8%) and highest in Syria (76.3%). Participants from all 13 countries exhibited significantly higher levels of coping compared to Libya (Table 5).

**Table 5.** Country-wise analyses for high psychological distress, fear of COVID-19, and coping among the study participants

Characteristics	K-10 Score				OR (univariable)	OR (multivariable)^a^
	coping (score 4–13) Low (score 10–15)	resilient coping (score 14–20) Moderate to very high (score 16–50)				
	*n*	%	*n*	%	OR (95% CI, *p* value)	OR (95% CI, *p* value)
Country of residence	802	32.8	1645	67.2		
UAE	8	1	26	1.4	Ref	Ref
Pakistan	21	2.6	42	2.6	0.62 (0.24–1.59, *p* = 0.316)	0.23 (0.05–0.99, *p* = 0.048)
Libya	24	3	56	3.3	0.72 (0.28–1.81, *p* = 0.483)	0.99 (0.23–4.33, *p* = 0.99)
Egypt	5	0.6	85	3.7	5.23 (1.57–17.38, *p* = 0.007)	2.67 (0.45–15.79, *p* = 0.28)
Palestine	7	0.9	88	3.9	3.87 (1.28–11.68, *p* = 0.016)	1.78 (0.36–8.81, *p* = 0.479)
Nepal	45	5.6	74	4.9	0.51 (0.21–1.21, *p* = 0.127)	0.29 (0.07–1.18, *p* = 0.083)
Kuwait	48	6	101	6.1	0.65 (0.27–1.54, *p* = 0.324)	0.57 (0.15–2.16, *p* = 0.412)
Oman	64	8	91	6.3	0.44 (0.19–1.03, *p* = 0.058)	0.42 (0.11–1.59, *p* = 0.199)
Syria	20	2.5	153	7.1	2.35 (0.94–5.9, *p* = 0.068)	1.95 (0.46–8.36, *p* = 0.368)
Saudi Arabia	79	9.9	168	10.1	0.65 (0.28–1.51, *p* = 0.32)	0.54 (0.14–2.01, *p* = 0.356)
Jordan	35	4.4	217	10.3	1.91 (0.8–4.55, *p* = 0.145)	1.40 (0.33–5.96, *p* = 0.647)
Hong Kong	123	15.3	159	11.5	0.40 (0.17–0.91, *p* = 0.029)	0.43 (0.11–1.67, *p* = 0.222)
Thailand	166	20.7	141	12.5	0.26 (0.11–0.6, *p* = 0.001)	0.27 (0.07–1.06, *p* = 0.06)
Indonesia	157	19.6	244	16.4	0.48 (0.21–1.08, *p* = 0.077)	0.44 (0.11–1.68, *p* = 0.229)
Characteristics	FCV-19S Score				OR (univariable)	OR (multivariable)
	Low (score 7–21)	High (score 22–35)				
	*n*	%	*n*	%	OR (95% CI, *p* value)	OR (95% CI, *p* value)
Country of residence	1960	80.1	486	19.9		
Libya	73	91.2	7	8.8	Ref	Ref
Thailand	272	88.6	35	11.4	1.34 (0.57–3.14, *p* = 0.498)	0.92 (0.33–2.56, *p* = 0.878)
UAE	30	88.2	4	11.8	1.39 (0.38–5.1, *p* = 0.619)	0.97 (0.22–4.27, *p* = 0.967)
Saudi Arabia	209	84.6	38	15.4	1.9 (0.81–4.43, *p* = 0.14)	1.1 (0.41–2.96, *p* = 0.849)
Jordan	210	83.3	42	16.7	2.09 (0.9–4.85, *p* = 0.087)	0.66 (0.22–1.98, *p* = 0.454)
Syria	143	83.1	29	16.9	2.11 (0.88–5.06, *p* = 0.092)	0.98 (0.35–2.73, *p* = 0.967)
Nepal	94	79	25	21	2.77 (1.14–6.77, *p* = 0.025)	1.94 (0.68–5.55, *p* = 0.216)
Palestine	75	78.9	20	21.1	2.78 (1.11–6.97, *p* = 0.029)	0.76 (0.24–2.42, *p* = 0.646)
Kuwait	117	78.5	32	21.5	2.85 (1.2–6.8, *p* = 0.018)	1.88 (0.67–5.23, *p* = 0.229)
Indonesia	307	76.6	94	23.4	3.19 (1.42–7.17, *p* = 0.005)	2.27 (0.89–5.75, *p* = 0.085)
Oman	118	76.1	37	23.9	3.27 (1.39–7.72, *p* = 0.007)	2.84 (1.01–8.01, *p* = 0.049)
Hong Kong	208	73.8	74	26.2	3.71 (1.64–8.42, *p* = 0.002)	2.98 (1.08–8.25, *p* = 0.035)
Egypt	61	67.8	29	32.2	4.96 (2.03–12.1, *p* < 0.001)	1.88 (0.63–5.62, *p* = 0.257)
Pakistan	43	68.3	20	31.7	4.85 (1.9–12.41, *p* < 0.001)	3.55 (1.16–10.86, *p* = 0.026)
Characteristics	BRCS Score				OR (univariable)	OR (multivariable)
	Low resilient coping (score 4–13)	Medium to High resilient coping (score 14–20)				
	*n*	%	*n*	%	OR (95% CI, *p* value)	OR (95% CI, *p* value)
Country of residence	904	37	1542	63		
Libya	57	72.2	22	27.8	Ref	Ref
Pakistan	35	55.6	28	44.4	2.07 (1.03–4.21, *p* = 0.041)	2.35 (0.99–5.62, *p* = 0.054)
Egypt	45	50	45	50	2.59 (1.37–4.99, *p* = 0.004)	3.91 (1.68–9.06, *p* = 0.001)
Saudi Arabia	110	44.5	137	55.5	3.23 (1.88–5.70, *p* < 0.001)	3.32 (1.65–6.66, *p* < 0.001)
Jordan	111	44	141	56	3.29 (1.92–5.81, *p* < 0.001)	5.2 (2.39–11.32, *p* < 0.001)
Hong Kong	116	41.1	166	58.9	3.71 (2.18–6.51, *p* < 0.001)	3.97 (1.88–8.41, *p* < 0.001)
Kuwait	57	38.3	92	61.7	4.18 (2.34–7.68, *p* < 0.001)	5.66 (2.66–12.04, *p* < 0.001)
UAE	12	35.3	22	64.7	4.75 (2.05–11.51, *p* < 0.001)	5.32 (1.73–16.38, *p* < 0.001)
Nepal	40	33.6	79	66.4	5.12 (2.78–9.68, *p* < 0.001)	4.68 (2.16–10.12, *p* < 0.001)
Palestine	31	32.6	64	67.4	5.35 (2.82–10.44, *p* < 0.001)	10.68 (4.5–25.32, *p* < 0.001)
Indonesia	119	29.7	282	70.3	6.14 (3.64–10.69, *p* < 0.001)	7.01 (3.59–13.66, *p* < 0.001)
Thailand	91	29.6	216	70.4	6.15 (3.60–10.84, *p* < 0.001)	4.27 (2.06–8.86, *p* < 0.001)
Oman	39	25.2	116	74.8	7.71 (4.24–14.45, *p* < 0.001)	8.92 (3.98–20, *p* < 0.001)
Syria	41	23.7	132	76.3	8.34 (4.62–15.53, *p* < 0.001)	11.88 (5.53–25.53, *p* < 0.001)

OR, odds ratio; CI, confidence interval.

a Adjusted for: age, gender, smoking, alcohol intake, living status, place of birth, country, education, employment status, employment stress, financial impact, contact with COVID-19 case, experience related to COVID-19, self-identification as a frontline or essential service worker, and healthcare service utilization.

## Discussion

### Key findings

Our study provided evidence relating to different factors associated with developing COVID-19-related psychological stress, fear, and coping among HCWs based on data from 14 countries across the globe. Findings indicated that factors, which were associated with both moderate to very-high levels of psychological stress and fear of COVID-19, were perceived distress related to employment status and comorbid conditions other than mental health issues. Moreover, higher levels of resilient coping were associated with being negatively impacted financially due to COVID-19, perceived mental health as good to excellent, and providing direct care to known/suspected COVID-19 cases.

Generally, those factors might be based on the nature of the work of HCWs, which implied that during the pandemic, they had been more frequently exposed to additional working hours and the increased risk of getting infected with COVID-19. For instance, psychological distress and fear of COVID-19 were emphasized in a previous study in Singapore, which included laboratory HCWs who were at a high risk of exposure to the SARS-CoV-2 virus from handling infected patients' blood samples, in addition to a marked increase in their workload (Teo *et al*., 2021). Increased risk of transmitting the infection to other family members was also a contributing factor. We also found a significant correlation between living with family members and a high rate of moderate to high psychological distress. Furthermore, a previous systematic review showed that psychological distress was significantly correlated with being at risk of coming into contact with infected patients (Sirois and Owens, 2020). This was also consistent with our findings as we also found that contact with infected/suspected cases was significantly correlated with moderate-to-high psychological distress levels; it suggested that HCWs became more worried about not only getting infected and/or transmitting it when dealing with COVID-19 cases, but also they could potentially transmit to their family members (Koh *et al*., 2005).

We also found that with or without testing positive for COVID-19, self-isolation was also correlated with fear of COVID-19, high psychological distress, and reduced coping scores. This might be attributed to the sense of isolation from the team, which could significantly affect the mental health of HCWs (Brooks *et al*., 2020; Rossi *et al*., 2020). We found that doctors were more frequently exposed to developing moderate to high psychological distress, which was consistent with previous studies (De Kock *et al*., 2021; Kafle *et al*., 2021). Nurses also reported high stress levels because they usually had more frequent contact with COVID-19 cases, higher workloads, and were more frequently females (Maunder *et al*., 2006). In our earlier investigation, we found that psychological distress was higher among doctors, with lower fear levels of COVID-19. Yet, medium to high coping levels were more frequent among nurses (Rahman *et al*., 2021*a*). In the present study, no significance was found regarding resilient coping and fear of COVID-19.

Interestingly, we found that high resilient coping was associated with negatively impacted financial situations secondary to COVID-19, providing direct care for COVID-19 cases, high levels of fear, and self-isolation. This was evident in a previous study where better mental health outcomes were correlated with better adaptive personality traits (Sirois and Owens, 2020). In this context, applying mental health check-ups could be considered a helpful tool that could provide mental health support resulting in reduction of stress among HCWs (Ito and Matsushima, 2017). Some studies have suggested that having a friendly authority to communicate experiences of psychological difficulties daily might be safer and more relaxing. There should be clear and transparent communication between healthcare facilities and HCWs to enable prompt referral to available psychiatric care or psychological counselling resources if required (Teo *et al*., 2021). It was also pertinent that HCWs should have received regular and accurate updates of the COVID-19 situation, which could assist them in pre-empting their potential workload and allay their fears and uncertainties pertaining to work to ensure better coping related to the HCWs' day-to-day activities (Teo *et al*., 2021). Taking into considerations the transcultural context within the participating countries, influence of social support, a frequently reported psychosocial resource of HCWs, was found to be a significant protective factor in terms of general mental health problems; that needs to be evaluated further and advocated to address both psychosocial stress and coping abilities.

Our findings also indicated wide variations in the burdens of psychological distress and fear of COVID-19 among HCWs. This could be explained in a number of ways: first, the relative quality of clinical care presented within healthcare systems was different among the countries participating in this study; second, limited resources could be more frequently associated with stress-related events; and third, more working hours could increase the risk of being infected, which significantly predisposed to developing psychological distress and fear of COVID-19 (Neto *et al*., 2020). All those issues reiterate that when designing specific interventions, HCWs should not be considered a homogenous population as psychological impact will vary across this category of population.

There were some limitations to the findings of our investigation. Firstly, a potential selection bias might be present resulting from conducting the study through an online survey that limited recruitment of eligible HCWs, not those using the internet. Secondly, we did not have information on how exactly each country's healthcare system was affected by the pandemic and whether the HCWs were overburdened or not. Thirdly, the study's cross-sectional design also restricted us from conducting a proper predictive analysis. However, it should be noted that as a result of the current strategies to encounter the COVID-19 pandemic, such as the use of telehealth and social distancing, this study design was the most suitable and applicable option for investigating that situation. In addition, we collected data from 14 countries with a large sample size, which added to the strength of the current investigation.

## Conclusion

Our study identified some important factors that could predict psychological distress and fear of COVID-19 and the level of resilient coping among HCWs. We found that age, perceived distress due to change of employment, co-morbidities other than mental health issues, perceived status of own mental health, being in contact with suspected/known cases, visiting a healthcare provider in the last 6 months for any reasons including COVID-related stress were significantly correlated with psychological distress, fear, and coping in this study. Therefore, it is most important to recognize the needs of HCWs and appreciate the factors that could significantly leverage their psychological wellbeing during such critical periods. Findings from this study might encourage healthcare authorities in different global communities to plan, develop, and implement adequate management strategies for HCWs, to support them psychologically during the critical period. The HCWs’ management plan should include an adequate supply of personal protective equipment, isolation of HCWs from their families during the infectious period, and development of an additional healthcare workforce to support the frontline HCWs in duties. Resilience and coping enhancement and/or fear and anxiety reduction programmes, in relation to COVID-19 impacts on employment/finance, health/illness conditions, and availability of appropriate healthcare services, could be considered. This intervention is highly important and essential specifically for those HCWs with moderate to high psychological distress and those with strong senses/feelings of self-isolation. Specifically with the ongoing pandemic along with emerging issues of new variants of COVID-19 which resulted in overwhelming burden on the HCWs, those measures could potentially reduce the frequency and severity of psychological wellbeing and improve the quality of care in clinical practice.

## Data Availability

All data generated or analysed during this study are included in this published article.

## Supplementary material

For supplementary material accompanying this paper visit https://doi.org/10.1017/gmh.2022.35. click here to view supplementary material
