# Supplementary material for: Psychological impact of COVID-19 on healthcare workers: cross-sectional analyses from 14 countries
Source: Glob Ment Health (Camb). 2022 Jul 8;9:328–38. doi: 10.1017/gmh.2022.35 (PMC9300980; doi:10.1017/gmh.2022.35)
Supplement: Supplementary file 1 [file S2054425122000358sup001.docx]

**Supplementary Table 1. Predictors for psychological distress among the study participants (based on the K-10 score)**

| **Characteristics** | **Low (Score 10-15)** | | | **Moderate to Very High (Score 16-50)** | | **OR (univariable)** | **OR (multivariable)** |
| --- | --- | --- | --- | --- | --- | --- | --- |
|  | **n** | **%** | **n** | | **%** | **OR (CI 95%, P-value)** | **OR (CI 95%, P-value)** |
| **Age groups** | **750** | **34.7** | **1409** | | **65.3** |  |  |
| 18-29 years | 160 | 20.4 | 623 | | 79.6 | Ref | Ref |
| 30-59 years | 522 | 40.6 | 765 | | 59.4 | 0.38 (0.31-0.46, p<0.001) | 0.47 (0.36-0.62, p<0.001) |
| 60 years and above | 68 | 76.4 | 21 | | 23.6 | 0.08 (0.05-0.13, p<0.001) | 0.13 (0.06-0.24, p<0.001) |
| **Gender** | **798** | **32.8** | **1637** | | **67.2** |  |  |
| Male | 236 | 32.5 | 490 | | 67.5 | Ref | Ref |
| Female | 562 | 32.9 | 1147 | | 67.1 | 0.98 (0.82-1.18, p=0.856) | 1.17 (0.89-1.54, p=0.269) |
| **Born in the same country of residence** | **788** | **32.6** | **1631** | | **67.4** |  |  |
| No | 102 | 32.8 | 209 | | 67.2 | Ref | Ref |
| Yes | 686 | 32.5 | 1422 | | 67.5 | 1.01 (0.78-1.30, p=0.929) | 0.87 (0.61-1.23, p=0.441) |
| **Living Status** | **792** | **32.7** | 1628 | | 67.3 |  |  |
| Without family members (on your own/shared house) | 142 | 38.6 | 226 | | 61.4 | Ref | Ref |
| With family members | 650 | 31.7 | 1402 | | 68.3 | 1.36 (1.08-1.70, p=0.009) | 1.57 (1.15-2.15, p=0.004) |
| **Highest educational/vocational qualification** | **790** | **32.6** | **1633** | | **67.4** |  |  |
| Grade 1 to 6/Primary | 2 | 40 | 3 | | 60 | Ref | Ref |
| Grade 7 to 12/Secondary/Higher Secondary/Intermedicate | 22 | 24.2 | 69 | | 75.8 | 2.09 (0.26-13.41, p=0.435) | 2.79 (0.23-41.37, p=0.415) |
| Certificate/Diploma/Trade qualification | 73 | 30.7 | 165 | | 69.3 | 1.51 (0.20-9.28, p=0.657) | 2.19 (0.20-29.95, p=0.518) |
| Bachelor/Masters/PhD | 693 | 33.2 | 1396 | | 66.8 | 1.34 (0.18-8.12, p=0.747) | 2.81 (0.26-37.72, p=0.389) |
| **Current employment condition** | **787** | **32.9** | **1607** | | **67.1** |  |  |
| Jobs affected by COVID-19 (lost job/workng hours reduced/afraid of job loss) | 683 | 35.8 | 1224 | | 64.2 | Ref | Ref |
| Have an income source (employed/Governent benefits) | 104 | 21.4 | 383 | | 78.6 | 2.05 (1.63-2.61, p<0.001) | 1.23 (0.88-1.71, p=0.223) |
| **Perceived distress due to change of employment status** | **766** | **32.5** | **1591** | | **67.5** |  |  |
| A little to None | 608 | 42.3 | 831 | | 57.7 | Ref | Ref |
| Moderate to A great deal | 158 | 17.2 | 760 | | 82.8 | 3.52 (2.89-4.31, p<0.001) | 2.16 (1.62-2.88, p<0.001) |
| **Improved working situation due to change of employment status** | **759** | **32.4** | **1586** | | **67.6** |  |  |
| A little to None | 593 | 34.2 | 1141 | | 65.8 | Ref | Ref |
| Moderate to A Great deal | 166 | 27.2 | 445 | | 72.8 | 1.39 (1.14-1.71, p=0.001) | 0.79 (0.59-1.05, p=0.098) |
| **Self-identification as a frontline or essential service worker** | **802** | **32.8** | **1645** | | **67.2** |  |  |
| No | 184 | 39.6 | 281 | | 60.4 | Ref | Ref |
| Yes | 618 | 31.2 | 1364 | | 68.8 | 1.45 (1.17-1.78, p=0.001) | 1.12 (0.83-1.51, p=0.462) |
| **Self-identification as a healthcare worker** | **802** | **32.8** | **1645** | | **67.2** |  |  |
| Yes, Doctor | 261 | 29.4 | 626 | | 70.6 | Ref | Ref |
| Yes, Nurse | 395 | 38.3 | 637 | | 61.7 | 0.67 (0.55-0.81, p<0.001) | 0.70 (0.53-0.93, p=0.014) |
| Yes, Other healthcare worker | 146 | 27.7 | 382 | | 72.3 | 1.09 (0.86-1.39, p=0.476) | 0.89 (0.63-1.26, p=0.502) |
| **COVID-19 impacted financial situation** | **802** | **32.8** | **1645** | | **67.2** |  |  |
| No impact | 443 | 40.4 | 653 | | 59.6 | Ref | Ref |
| Yes, impacted positively | 89 | 29.1 | 217 | | 70.9 | 1.65 (1.26-2.18, p<0.001) | 1.08 (0.74-1.59, p=0.691) |
| Yes, impacted negatively | 270 | 25.8 | 775 | | 74.2 | 1.95 (1.62-2.34, p<0.001) | 0.71 (0.53-0.96, p=0.026) |
| **Affected by the change in financial situation** | **781** | **32.5** | **1621** | | **67.5** |  |  |
| Not at all | 267 | 50.8 | 259 | | 49.2 | Ref | Ref |
| Unsure at this time | 146 | 36.2 | 257 | | 63.8 | 1.81 (1.39-2.37, p<0.001) | 1.53 (1.07-2.18, p=0.019) |
| Somewhat | 298 | 27.1 | 800 | | 72.9 | 2.77 (2.23-3.44, p<0.001) | 1.94 (1.41-2.69, p<0.001) |
| A great extent | 70 | 18.7 | 305 | | 81.3 | 4.49 (3.31-6.16, p<0.001) | 2.10 (1.33-3.36, p=0.002) |
| **Co-morbidities** | **802** | **32.8** | **1645** | | **67.2** |  |  |
| No comorbid conditions | 613 | 34.4 | 1167 | | 65.6 | Ref | Ref |
| Psychiatric/Mental health issues | 3 | 4.5 | 63 | | 95.5 | 11.03 (4.07-45.27, p | 3.09 (0.85-20.08, p=0.141) |
| Other comorbid conditions | 186 | 31 | 414 | | 69 | 1.17 (0.96-1.43, p=0.123) | 1.35 (1.02-1.79, p=0.038) |
| **Co-morbidities** | **802** | **32.8** | **1645** | | **67.2** |  | Not included in multivariate model |
| No comorbid conditions | 613 | 34.4 | 1167 | | 65.6 | Ref |  |
| Single comorbid condition | 136 | 27.5 | 358 | | 72.5 | 1.38 (1.11-1.73, p=0.004) |  |
| Multiple comorbid conditions | 53 | 30.8 | 119 | | 69.2 | 1.18 (0.85-1.67, p=0.339) |  |
| **Perceived status of own mental health** | **802** | **32.8** | **1645** | | **67.2** |  |  |
| Poor to Fair | 54 | 8.8 | 562 | | 91.2 | Ref | Ref |
| Good to Excellent | 748 | 40.9 | 1083 | | 59.1 | 0.14 (0.10-0.19, p | 0.17 (0.11-0.24, p<0.001) |
| **Smoking** | **802** | **32.8** | **1645** | | **67.2** |  |  |
| Never smoker | 717 | 34.3 | 1371 | | 65.7 | Ref | Ref |
| Ever smoker | 85 | 23.7 | 274 | | 76.3 | 1.69 (1.31-2.20, p | 1.47 (1.00-2.17, p=0.049) |
| **Increased smoking over the last 6 months** | **59** | **22** | **209** | | **78** |  | Not included in multivariate model |
| No | 36 | 27.5 | 95 | | 72.5 | Ref |  |
| Yes | 23 | 16.8 | 114 | | 83.2 | 1.88 (1.05-3.42, p=0.036) |  |
| **Current alcohol drinking (last 4 weeks)** | **784** | **32.6** | **1621** | | **67.4** |  |  |
| No | 715 | 33.1 | 1444 | | 66.9 | Ref | Ref |
| Yes | 69 | 28 | 177 | | 72 | 1.27 (0.95-1.71, p=0.109) | 1.11 (0.76-1.64, p=0.575) |
| **Increased alcohol drinking over the last 6 months** | **69** | **28** | **177** | | **72** |  | Not included in multivariate model |
| No | 61 | 34.1 | 118 | | 65.9 | Ref |  |
| Yes | 8 | 11.9 | 59 | | 88.1 | 3.81 (1.80-9.10, p=0.001) |  |
| **Contact with known/suspected cases of COVID-19** | **784** | **32.5** | **1627** | | **67.5** |  |  |
| No | 404 | 39.6 | 617 | | 60.4 | Ref | Ref |
| Unsure | 55 | 22.2 | 193 | | 77.8 | 2.30 (1.67-3.20, p<0.001) | 1.93 (1.27-2.97, p=0.002) |
| Yes, had indirect contact | 108 | 27.8 | 281 | | 72.2 | 1.70 (1.32-2.20, p<0.001) | 1.42 (1.02-1.99, p=0.039) |
| Yes, provided direct care | 217 | 28.8 | 536 | | 71.2 | 1.62 (1.32-1.98, p<0.001) | 1.21 (0.89-1.64, p=0.227) |
| **Experience related to COVID-19 pandemic** | **771** | **32.4** | **1606** | | **67.6** |  |  |
| No known exposure to COVID-19 | 561 | 35 | 1042 | | 65 | Ref | Ref |
| Tested positive for COVID-19 | 74 | 27 | 200 | | 73 | 1.46 (1.10-1.95, p=0.010) | 0.87 (0.58-1.31, p=0.489) |
| Tested negative for COVID-19 but self-isolating | 127 | 27.5 | 334 | | 72.5 | 1.42 (1.13-1.78, p=0.003) | 1.07 (0.78-1.47, p=0.667) |
| Recent overseas travel history and was in quarantine | 9 | 23.1 | 30 | | 76.9 | 1.79 (0.88-4.04, p=0.127) | 1.29 (0.51-3.49, p=0.602) |
| **Self-identification as a patient (visited a healthcare provider in the last 6 months)** | **788** | **32.7** | **1622** | | **67.3** |  |  |
| No | 568 | 36.8 | 974 | | 63.2 | Ref | Ref |
| Yes | 220 | 25.3 | 648 | | 74.7 | 1.72 (1.43-2.07, p<0.001) | 1.57 (1.22-2.03, p=0.001) |
| **Types of healthcare service used** | **192** | **24.6** | **589** | | **75.4** |  | Not included in multivariate model |
| Visited a healthcare provider in person | 161 | 27.2 | 432 | | 72.8 | Ref |  |
| Telehealth consultation/Used healpline | 27 | 18.9 | 116 | | 81.1 | 1.60 (1.03-2.57, p=0.043) |  |
| Used both | 4 | 8.9 | 41 | | 91.1 | 3.82 (1.51-12.86, p=0.012) |  |
| **Level of fear of COVID-19 (FCV-19S categories)** | **802** | **32.8** | **1645** | | **67.2** |  |  |
| Low (score 10-15) | 743 | 37.9 | 1217 | | 62.1 | Ref | Ref |
| Moderate to Very High (score 16-50) | 59 | 12.1 | 427 | | 87.9 | 4.42 (3.34-5.94, p<0.001) | 3.36 (2.37-4.85, p<0.001) |
| **Level of coping (BRCS categories)** | **801** | **32.7** | **1645** | | **67.3** |  |  |
| Low (Score 7-21) | 249 | 27.5 | 655 | | 72.5 | Ref | Ref |
| High (Score 22-35) | 552 | 35.8 | 990 | | 64.2 | 0.68 (0.57-0.81, p<0.001) | 0.84 (0.66-1.07, p=0.163) |
| **Healthcare service use to overcome COVID-19 related stress in the last 6 months** | **781** | **32.5** | **1621** | | **67.5** |  |  |
| No | 744 | 36 | 1324 | | 64 | Ref | Ref |
| Yes | 37 | 11 | 298 | | 89 | 4.53 (3.22-6.54, p<0.001) | 2.81 (1.75-4.65, p<0.001) |

OR: Odds ratio; CI: Confidence interval

**Supplementary Table 2. Predictors for fear of COVID-19 among the study participants (based on the FCV-19S score)**

| **Characteristics** | Low (Score 7-21) | | | High (Score 22-35) | | **OR (univariable)** | **OR (multivariable)** |
| --- | --- | --- | --- | --- | --- | --- | --- |
|  | **n** | **%** | **n** | | **%** | **OR (CI 95%, P-value)** | **OR (CI 95%, P-value)** |
| **Age groups** | **1729** | **80.1** | **429** | | **19.9** |  |  |
| 18-29 years | 632 | 80.8 | 150 | | 19.2 | Ref | Ref |
| 30-59 years | 1021 | 79.3 | 266 | | 20.7 | 1.10 (0.88-1.37, p=0.413) | 1.48 (1.12-1.97, p=0.007) |
| 60 years and above | 76 | 85.4 | 13 | | 14.6 | 0.72 (0.37-1.29, p=0.296) | 2.15 (0.94-4.54, p=0.055) |
| **Gender** | **1949** | **80.1** | **485** | | **19.9** |  |  |
| Male | 610 (84.1) | 84.1 | 115 | | 15.9 | Ref | Ref |
| Female | 1339 (78.3) | 78.3 | 370 | | 21.7 | 1.47 (1.17-1.85, p=0.001) | 1.55 (1.14-2.11, p=0.005) |
| **Born in the same country of residence** | **1933** | **79.9** | **485** | | **20.1** |  |  |
| No | 241 | 77.5 | 70 | | 22.5 | Ref | Ref |
| Yes | 1692 | 80.3 | 415 | | 19.7 | 0.84 (0.64-1.13, p=0.248) | 0.72 (0.50-1.05, p=0.085) |
| **Living Status** | 1936 | **80** | **483** | | **20** |  |  |
| Without family members (on your own/shared house) | 296 | 80.4 | 72 | | 19.6 | Ref | Ref |
| With family members | 1640 | 80 | 411 | | 20 | 1.03 (0.78-1.37, p=0.834) | 1.05 (0.75-1.51, p=0.767) |
| **Highest educational/vocational qualification** | **1938** | **80** | **484** | | **20** |  |  |
| Grade 1 to 6/Primary | 4 | 80 | 1 | | 20 | Ref | Ref |
| Grade 7 to 12/Secondary/Higher Secondary/Intermedicate | 72 | 79.1 | 19 | | 20.9 | 1.06 (0.15-21.29, p=0.962) | 0.75 (0.07-17.44, p=0.823) |
| Certificate/Diploma/Trade qualification | 168 | 70.6 | 70 | | 29.4 | 1.67 (0.24-32.91, p=0.650) | 1.36 (0.15-30.17, p=0.801) |
| Bachelor/Masters/PhD | 1694 | 81.1 | 394 | | 18.9 | 0.93 (0.14-18.23, p=0.949) | 0.79 (0.09-17.31, p=0.849) |
| **Current employment condition** | **1918** | **80.2** | **475** | | **19.8** |  |  |
| Jobs affected by COVID-19 (lost job/workng hours reduced/afraid of job loss) | 1554 | 81.5 | 352 | | 18.5 | Ref | Ref |
| Have an income source (employed/Governent benefits) | 364 | 74.7 | 123 | | 25.3 | 1.49 (1.18-1.88, p=0.001) | 1.06 (0.77-1.46, p=0.700) |
| **Perceived distress due to change of employment status** | **1882** | **79.9** | **474** | | **20.1** |  |  |
| A little to None | 1233 | 85.7 | 205 | | 14.3 | Ref | Ref |
| Moderate to A great deal | 649 | 70.7 | 269 | | 29.3 | 2.49 (2.03-3.06, p<0.001) | 1.87 (1.41-2.48, p<0.001) |
| **Improved working situation due to change of employment status** | **1868** | **79.7** | **476** | | **20.3** |  |  |
| A little to None | 1422 | 82.1 | 311 | | 17.9 | Ref | Ref |
| Moderate to A Great deal | 446 | 73 | 165 | | 27 | 1.69 (1.36-2.10, p<0.001) | 1.30 (0.98-1.72, p=0.069) |
| **Self-identification as a frontline or essential service worker** | **1960** | **80.1** | **486** | | **19.9** |  |  |
| No | 392 | 84.3 | 73 | | 15.7 | Ref | Ref |
| Yes | 1568 | 79.2 | 413 | | 20.8 | 1.41 (1.08-1.87, p=0.013) | 1.40 (0.99-2.00, p=0.059) |
| **Self-identification as a healthcare worker** | **1960** | **80.1** | **486** | | **19.9** |  |  |
| Yes, Doctor | 712 | 80.4 | 174 | | 19.6 | Ref | Ref |
| Yes, Nurse | 838 | 81.2 | 194 | | 18.8 | 0.95 (0.75-1.19, p=0.641) | 1.03 (0.76-1.40, p=0.850) |
| Yes, Other healthcare worker | 410 | 77.7 | 118 | | 22.3 | 1.18 (0.90-1.53, p=0.224) | 1.23 (0.87-1.74, p=0.245) |
| **COVID-19 impacted financial situation** | **1960** | **80.1** | **486** | | **19.9** |  |  |
| No impact | 925 | 84.4 | 171 | | 15.6 | Ref | Ref |
| Yes, impacted positively | 235 | 76.8 | 71 | | 23.2 | 1.63 (1.19-2.22, p=0.002) | 1.30 (0.87-1.93, p=0.195) |
| Yes, impacted negatively | 800 | 76.6 | 244 | | 23.4 | 1.65 (1.33-2.05, p<0.001) | 1.16 (0.83-1.61, p=0.383) |
| **Affected by the change in financial situation** | **1921** | **80** | **480** | | **20** |  |  |
| Not at all | 458 | 87.1 | 68 | | 12.9 | Ref | Ref |
| Unsure at this time | 327 | 81.1 | 76 | | 18.9 | 1.57 (1.10-2.24, p=0.014) | 1.06 (0.68-1.64, p=0.808) |
| Somewhat | 874 | 79.7 | 223 | | 20.3 | 1.72 (1.29-2.32, p<0.001) | 1.13 (0.75-1.70, p=0.565) |
| A great extent | 262 | 69.9 | 113 | | 30.1 | 2.90 (2.08-4.08, p<0.001) | 1.15 (0.71-1.86, p=0.582) |
| **Co-morbidities** | **1959** | **80.1** | **486** | | **19.9** |  |  |
| No comorbid conditions | 1459 | 82 | 320 | | 18 | Ref | Ref |
| Psychiatric/Mental health issues | 49 | 74.2 | 17 | | 25.8 | 1.58 (0.88-2.73, p=0.112) | 0.50 (0.22-1.05, p=0.081) |
| Other comorbid conditions | 451 | 75.2 | 149 | | 24.8 | 1.51 (1.21-1.88, p<0.001) | 1.45 (1.09-1.92, p=0.010) |
| **Co-morbidities** | **1959** | **80.1** | **486** | | **19.9** |  | Not included in multivariate model |
| No comorbid conditions | 1459 | 82 | 320 | | 18 | Ref |  |
| Single comorbid condition | 377 | 76.3 | 117 | | 23.7 | 1.41 (1.11-1.79, p=0.005) |  |
| Multiple comorbid conditions | 123 | 71.5 | 49 | | 28.5 | 1.82 (1.27-2.57, p=0.001) |  |
| **Perceived status of own mental health** | **1960** | **80.1** | **486** | | **19.9** |  |  |
| Poor to Fair | 427 | 69.3 | 189 | | 30.7 | Ref | Ref |
| Good to Excellent | 1533 | 83.8 | 297 | | 16.2 | 0.44 (0.35-0.54, p<0.001) | 0.72 (0.55-0.94, p=0.017) |
| **Smoking** | **1960** | **80.1** | **486** | | **19.9** |  |  |
| Never smoker | 1668 | 79.9 | 419 | | 20.1 | Ref | Ref |
| Ever smoker | 292 | 81.3 | 67 | | 18.7 | 0.91 (0.68-1.21, p=0.535) | 0.83 (0.56-1.21, p=0.334) |
| **Increased smoking over the last 6 months** | **219** | **81.7** | **49** | | **18.3** |  | Not included in multivariate model |
| No | 109 | 83.2 | 22 | | 16.8 | Ref |  |
| Yes | 110 | 80.3 | 27 | | 19.7 | 1.22 (0.65-2.28, p=0.538) |  |
| **Current alcohol drinking (last 4 weeks)** | **1922** | **80** | **482** | | **20** |  |  |
| No | 1742 | 80.7 | 416 | | 19.3 | Ref | Ref |
| Yes | 180 | 73.2 | 66 | | 26.8 | 1.54 (1.13-2.07, p=0.005) | 1.68 (1.16-2.41, p=0.006) |
| **Increased alcohol drinking over the last 6 months** | **180** | **73.2** | **66** | | **26.8** |  | Not included in multivariate model |
| No | 143 | 79.9 | 36 | | 20.1 | Ref |  |
| Yes | 37 | 55.2 | 30 | | 44.8 | 3.22 (1.76-5.92, p<0.001) |  |
| **Contact with known/suspected cases of COVID-19** | **1926** | **79.9** | **484** | | **20.1** |  |  |
| No | 845 | 82.8 | 176 | | 17.2 | Ref | Ref |
| Unsure | 175 | 70.6 | 73 | | 29.4 | 2.00 (1.45-2.74, p<0.001) | 1.69 (1.14-2.48, p=0.008) |
| Yes, had indirect contact | 302 | 77.6 | 87 | | 22.4 | 1.38 (1.03-1.84, p=0.028) | 1.08 (0.75-1.54, p=0.666) |
| Yes, provided direct care | 604 | 80.3 | 148 | | 19.7 | 1.18 (0.92-1.50, p=0.189) | 0.89 (0.63-1.24, p=0.485) |
| **Experience related to COVID-19 pandemic** | **1898** | **79.9** | **478** | | **20.1** |  |  |
| No known exposure to COVID-19 | 1314 | 82 | 288 | | 18 | Ref | Ref |
| Tested positive for COVID-19 | 220 | 80.3 | 54 | | 19.7 | 1.12 (0.80-1.54, p=0.493) | 1.04 (0.67-1.59, p=0.867) |
| Tested negative for COVID-19 but self-isolating | 337 | 73.1 | 124 | | 26.9 | 1.68 (1.32-2.14, p<0.001) | 1.19 (0.86-1.63, p=0.293) |
| Recent overseas travel history and was in quarantine | 27 | 69.2 | 12 | | 30.8 | 2.03 (0.98-3.96, p=0.045) | 1.16 (0.49-2.58, p=0.729) |
| **Self-identification as a patient (visited a healthcare provider in the last 6 months)** | **1926** | **80** | **483** | | **20** |  |  |
| No | 1236 | 80.2 | 305 | | 19.8 | Ref | Ref |
| Yes | 690 | 79.5 | 178 | | 20.5 | 1.05 (0.85-1.28, p=0.674) | 0.76 (0.58-0.99, p=0.046) |
| **Types of healthcare service used** | **612** | **78.4** | **169** | | **21.6** |  | Not included in multivariate model |
| Visited a healthcare provider in person | 474 | 79.9 | 119 | | 20.1 | Ref |  |
| Telehealth consultation/Used healpline | 98 | 68.5 | 45 | | 31.5 | 1.83 (1.21-2.73, p=0.004) |  |
| Used both | 40 | 88.9 | 5 | | 11.1 | 0.50 (0.17-1.18, p=0.151) |  |
| **Level of psychological distress (K10 categories)** | **1960** | **80.1** | **486** | | **19.9** |  |  |
| Low (score 10-15) | 743 | 92.6 | 59 | | 7.4 | Ref | Ref |
| Moderate to Very High (score 16-50) | 1217 | 74 | 427 | | 26 | 4.42 (3.34-5.94, p<0.001) | 3.43 (2.44-4.92, p<0.001) |
| **Level of coping (BRCS categories)** | **1959** | **80.1** | **486** | | **19.9** |  |  |
| Low (Score 7-21) | 698 | 77.2 | 206 | | 22.8 | Ref | Ref |
| High (Score 22-35) | 1261 | 81.8 | 280 | | 18.2 | 0.75 (0.61-0.92, p=0.006) | 0.77 (0.59-0.99, p=0.040) |
| **Healthcare service use to overcome COVID-19 related stress in the last 6 months** | **1921** | **80** | **481** | | **20** |  |  |
| No | 1707 | 82.6 | 360 | | 17.4 | Ref | Ref |
| Yes | 214 | 63.9 | 121 | | 36.1 | 2.68 (2.08-3.44, p<0.001 | 1.94 (1.38-2.72, p |

OR: Odds ratio; CI: Confidence interval

**Supplementary Table 3. Predictors for coping among the study participants (based on the BRCS score)**

| **Characteristics** | **Low resilient copers (Score 4-13)** | | **Medium to High resilient copers (Score 14-20)** | | **OR (univariable)** | **OR (multivariable)** |
| --- | --- | --- | --- | --- | --- | --- |
|  | **n** | **%** | **n** | **%** | **OR (CI 95%, P-value)** | **OR (CI 95%, P-value)** |
| **Age groups** | **771** | **35.7** | **1388** | **64.3** |  |  |
| 18-29 years | 307 | 39.2 | 476 | 60.8 | Ref | Ref |
| 30-59 years | 438 | 34 | 849 | 66 | 1.25 (1.04-1.50, p=0.017) | 1.31 (1.04-1.65, p=0.020) |
| 60 years and above | 26 | 29.2 | 63 | 70.8 | 1.56 (0.98-2.56, p=0.068) | 1.39 (0.79-2.52, p=0.262) |
| **Gender** | **899** | **36.9** | **1535** | **63.1** |  |  |
| Male | 297 | 41 | 428 | 59 | Ref | Ref |
| Female | 602 | 35.2 | 1107 | 64.8 | 1.28 (1.07-1.52, p=0.007) | 1.17 (0.92-1.48, p=0.199) |
| **Born in the same country of residence** | **889** | **36.8** | **1529** | **63.2** |  |  |
| No | 109 | 35 | 202 | 65 | Ref | Ref |
| Yes | 780 | 37 | 1327 | 63 | 0.92 (0.71-1.18, p=0.501) | 0.98 (0.72-1.33, p=0.914) |
| **Living Status** | **887** | **36.7** | **1532** | **63.3** |  |  |
| Without family members (on your own/shared house) | 127 | 34.5 | 241 | 65.5 | Ref | Ref |
| With family members | 760 | 37.1 | 1291 | 62.9 | 0.90 (0.71-1.13, p=0.351) | 0.98 (0.74-1.29, p=0.859) |
| **Highest educational/vocational qualification** | **886** | **36.6** | **1536** | **63.4** |  |  |
| Grade 1 to 6/Primary | 4 | 80 | 1 | 20 | Ref | Ref |
| Grade 7 to 12/Secondary/Higher Secondary/Intermedicate | 39 | 42.9 | 52 | 57.1 | 5.33 (0.75-106.55, p=0.141) | 5.02 (0.56-108.54, p=0.184) |
| Certificate/Diploma/Trade qualification | 110 | 46.2 | 128 | 53.8 | 4.65 (0.68-91.78, p=0.172) | 4.61 (0.55-96.82, p=0.199) |
| Bachelor/Masters/PhD | 733 | 35.1 | 1355 | 64.9 | 7.39 (1.09-144.79, p=0.074) | 6.55 (0.79-136.26, p=0.111) |
| **Current employment condition** | **876** | **36.6** | **1517** | **63.4** |  |  |
| Jobs affected by COVID-19 (lost job/workng hours reduced/afraid of job loss) | 684 | 35.9 | 1222 | 64.1 | Ref | Ref |
| Have an income source (employed/Governent benefits) | 192 | 39.4 | 295 | 60.6 | 0.86 (0.70-1.06, p=0.148) | 1.11 (0.85-1.47, p=0.435) |
| **Perceived distress due to change of employment status** | **870** | **36.9** | **1486** | **63.1** |  |  |
| A little to None | 510 | 35.5 | 928 | 64.5 | Ref | Ref |
| Moderate to A great deal | 360 | 39.2 | 558 | 60.8 | 0.85 (0.72-1.01, p=0.066) | 0.82 (0.64-1.04, p=0.099) |
| **Improved working situation due to change of employment status** | **861** | **36.7** | **1483** | **63.3** |  |  |
| A little to None | 639 | 36.9 | 1094 | 63.1 | Ref | Ref |
| Moderate to A Great deal | 222 | 36.3 | 389 | 63.7 | 1.02 (0.85-1.24, p=0.812) | 1.18 (0.93-1.51, p=0.176) |
| **Self-identification as a frontline or essential service worker** | **904** | **37** | **1542** | **63** |  |  |
| No | 167 | 35.9 | 298 | 64.1 | Ref | Ref |
| Yes | 737 | 37.2 | 1244 | 62.8 | 0.95 (0.77-1.17, p=0.604) | 0.94 (0.72-1.22, p=0.629) |
| **Self-identification as a healthcare worker** | **904** | **37** | **1542** | **63** |  |  |
| Yes, Doctor | 331 | 37.4 | 555 | 62.6 | Ref | Ref |
| Yes, Nurse | 371 | 35.9 | 661 | 64.1 | 1.06 (0.88-1.28, p=0.523) | 1.05 (0.82-1.34, p=0.703) |
| Yes, Other healthcare worker | 202 | 38.3 | 326 | 61.7 | 0.96 (0.77-1.20, p=0.736) | 1.09 (0.82-1.45, p=0.570) |
| **COVID-19 impacted financial situation** | **904** | **37** | **1542** | **63** |  |  |
| No impact | 422 | 38.5 | 673 | 61.5 | Ref | Ref |
| Yes, impacted positively | 129 | 42.2 | 177 | 57.8 | 0.86 (0.67-1.11, p=0.252) | 1.10 (0.79-1.52, p=0.580) |
| Yes, impacted negatively | 353 | 33.8 | 692 | 66.2 | 1.23 (1.03-1.47, p=0.022) | 1.75 (1.34-2.27, p<0.001) |
| **Affected by the change in financial situation** | **885** | **36.9** | **1516** | **63.1** |  |  |
| Not at all | 187 | 35.6 | 339 | 64.4 | Ref | Ref |
| Unsure at this time | 167 | 41.4 | 236 | 58.6 | 0.78 (0.60-1.02, p=0.067) | 0.76 (0.55-1.04, p=0.087) |
| Somewhat | 386 | 35.2 | 711 | 64.8 | 1.02 (0.82-1.26, p=0.886) | 0.93 (0.69-1.26, p=0.653) |
| A great extent | 145 | 38.7 | 230 | 61.3 | 0.87 (0.67-1.15, p=0.339) | 0.84 (0.57-1.24, p=0.386) |
| **Co-morbidities** | **903** | **36.9** | **1542** | **63.1** |  |  |
| No comorbid conditions | 639 | 35.9 | 1140 | 64.1 | Ref | Ref |
| Psychiatric/Mental health issues | 38 | 57.6 | 28 | 42.4 | 0.41 (0.25-0.68, p<0.001) | 0.48 (0.26-0.90, p=0.023) |
| Other comorbid conditions | 226 | 37.7 | 374 | 62.3 | 0.93 (0.77-1.12, p=0.442) | 1.00 (0.79-1.28, p=0.976) |
| **Co-morbidities** | **903** | **36.9** | **1542** | **63.1** |  | Not included in multivariate model |
| No comorbid conditions | 639 | 35.9 | 1140 | 64.1 | Ref |  |
| Single comorbid condition | 210 | 42.5 | 284 | 57.5 | 0.76 (0.62-0.93, p=0.007) |  |
| Multiple comorbid conditions | 54 | 31.4 | 118 | 68.6 | 1.22 (0.88-1.73, p=0.237) |  |
| **Perceived status of own mental health** | **904** | **37** | **1542** | **63** |  |  |
| Poor to Fair | 285 | 46.3 | 331 | 53.7 | Ref | Ref |
| Good to Excellent | 619 | 33.8 | 1211 | 66.2 | 1.68 (1.40-2.03, p<0.001) | 1.48 (1.17-1.88, p=0.001) |
| **Smoking** | **904** | **37** | **1542** | **63** |  |  |
| Never smoker | 750 | 35.9 | 1338 | 64.1 | Ref | Ref |
| Ever smoker | 154 | 43 | 204 | 57 | 0.74 (0.59-0.93, p=0.010) | 1.02 (0.75-1.39, p=0.906) |
| **Increased smoking over the last 6 months** | **116** | **43.4** | **151** | **56.6** |  | Not included in multivariate model |
| No | 60 | 45.8 | 71 | 54.2 | Ref |  |
| Yes | 56 | 41.2 | 80 | 58.8 | 1.21 (0.74-1.96, p=0.446) |  |
| **Current alcohol drinking (last 4 weeks)** | **881** | **36.6** | **1523** | **63.4** |  |  |
| No | 773 | 35.8 | 1385 | 64.2 | Ref | Ref |
| Yes | 108 | 43.9 | 138 | 56.1 | 0.71 (0.55-0.93, p=0.013) | 0.82 (0.60-1.12, p=0.204) |
| **Increased alcohol drinking over the last 6 months** | **108** | **43.9** | **138** | **56.1** |  | Not included in multivariate model |
| No | 69 | 38.5 | 110 | 61.5 | Ref |  |
| Yes | 39 | 58.2 | 28 | 41.8 | 0.45 (0.25-0.79, p=0.006) |  |
| **Contact with known/suspected cases of COVID-19** | **882** | **36.6** | **1528** | **63.4** |  |  |
| No | 392 | 38.4 | 629 | 61.6 | Ref | Ref |
| Unsure | 108 | 43.5 | 140 | 56.5 | 0.81 (0.61-1.07, p=0.137) | 0.94 (0.68-1.32, p=0.725) |
| Yes, had indirect contact | 134 | 34.5 | 254 | 65.5 | 1.18 (0.93-1.51, p=0.181) | 1.25 (0.93-1.68, p=0.149) |
| Yes, provided direct care | 248 | 32.9 | 505 | 67.1 | 1.27 (1.04-1.55, p=0.018) | 1.47 (1.12-1.92, p=0.005) |
| **Experience related to COVID-19 pandemic** | **866** | **36.4** | **1510** | **63.6** |  |  |
| No known exposure to COVID-19 | 568 | 35.4 | 1035 | 64.6 | Ref | Ref |
| Tested positive for COVID-19 | 91 | 33.2 | 183 | 66.8 | 1.10 (0.84-1.45, p=0.477) | 1.01 (0.71-1.45, p=0.965) |
| Tested negative for COVID-19 but self-isolating | 185 | 40.2 | 275 | 59.8 | 0.82 (0.66-1.01, p=0.061) | 0.76 (0.58-0.99, p=0.039) |
| Recent overseas travel history and was in quarantine | 22 | 56.4 | 17 | 43.6 | 0.42 (0.22-0.80, p=0.009) | 0.58 (0.28-1.19, p=0.136) |
| **Self-identification as a patient (visited a healthcare provider in the last 6 months)** | **881** | **36.6** | **1528** | **63.4** |  |  |
| No | 575 | 37.3 | 967 | 62.7 | - | Ref |
| Yes | 306 | 35.3 | 561 | 64.7 | 1.09 (0.92-1.30, p=0.329) | 1.22 (0.98-1.51, p=0.080) |
| **Types of healthcare service used** | **267** | **34.2** | **514** | **65.8** |  | Not included in multivariate mode |
| Visited a healthcare provider in person | 197 | 33.2 | 396 | 66.8 | Ref |  |
| Telehealth consultation/Used healpline | 59 | 41.3 | 84 | 58.7 | 0.71 (0.49-1.03, p=0.071) |  |
| Used both | 11 | 24.4 | 34 | 75.6 | 1.54 (0.79-3.24, p=0.229) |  |
| **Level of psychological distress (K10 categories)** | **904** | **37** | **1541** | **63** |  |  |
| Low (score 10-15) | 249 | 31.1 | 552 | 68.9 | Ref | Ref |
| Moderate to Very High (score 16-50) | 655 | 39.8 | 990 | 60.2 | 0.68 (0.57-0.81, p | 0.84 (0.66-1.07, p=0.157) |
| **Level of fear of COVID-19 (FCV-19S categories)** | **875** | **36.4** | **1528** | **63.6** |  |  |
| Low (Score 7-21) | 698 | 35.6 | 1261 | 64.4 | Ref | Ref |
| High (Score 22-35) | 206 | 42.4 | 280 | 57.6 | 0.75 (0.61-0.92, p=0.006) | 0.75 (0.59-0.97, p=0.028) |
| **Healthcare service use to overcome COVID-19 related stress in the last 6 months** | **875** | **36.4** | **1528** | **63.6** |  |  |
| No | 732 | 35.4 | 1336 | 64.6 | Ref | Ref |
| Yes | 143 | 42.7 | 192 | 57.3 | 0.74 (0.58-0.93, p=0.010) | 0.86 (0.63-1.18, p=0.348) |

OR: Odds ratio; CI: Confidence interval
